# Absence of Rac1 and Rac3 GTPases in the nervous system hinders thymic, splenic and immune-competence development

**DOI:** 10.1002/eji.201040892

**Published:** 2011-03-07

**Authors:** Veronica Basso, Sara Corbetta, Sara Gualdoni, Diletta Tonoli, Pietro Luigi Poliani, Francesca Sanvito, Claudio Doglioni, Anna Mondino, Ivan de Curtis

**Affiliations:** 1 Lymphocyte Activation Unit, Division of Immunology, Transplantation and Infectious Diseases, San Raffaele Scientific InstituteMilan, Italy; 2 Cell Adhesion Unit, Division of Neuroscience, San Raffaele Scientific InstituteMilan, Italy; 3 Department of Pathology, University of Brescia Medical SchoolBrescia, Italy; 4 Pathology Unit, San Raffaele Scientific InstituteMilan, Italy; 5 Vita-Salute San Raffaele UniversityMilan, Italy

**Keywords:** Immune-competence, Neuroimmunology, Neurons, Rac GTPases, Thymus

## Abstract

The nervous system influences organ development by direct innervation and the action of hormones. We recently showed that the specific absence of Rac1 in neurons (Rac1^N^) in a Rac3-deficient (Rac3^KO^) background causes motor behavioural defects, epilepsy, and premature mouse death around postnatal day 13. We report here that Rac1^N^/Rac3^KO^ mice display a progressive loss of immune-competence. Comparative longitudinal analysis of lymphoid organs from control, single Rac1^N^ or Rac3^KO^, and double Rac1^N^/Rac3^KO^ mutant animals showed that thymus development is preserved up to postnatal day 9 in all animals, but is impaired in Rac1^N^/Rac3^KO^ mice at later times. This is evidenced by a drastic reduction in thymic cell numbers. Cell numbers were also reduced in the spleen, leading to splenic tissue disarray. Organ involution occurs in spite of unaltered thymocyte and lymphocyte subset composition, and proper mature T-cell responses to polyclonal stimuli in vitro. Suboptimal thymus innervation by tau-positive neuronal terminals possibly explains the suboptimal thymic output and arrested thymic development, which is accompanied by higher apoptotic rates. Our results support a role for neuronal Rac1 and Rac3 in dictating proper lymphoid organ development, and suggest the existence of lymphoid-extrinsic mechanisms linking neural defects to the loss of immune-competence.

## Introduction

Over the past several years complex bidirectional interactions between the nervous system and the immune system have been identified, which seem necessary to maintain homeostasis in both systems and to regulate the immune response. Sympathetic innervation of lymphoid organs is involved in the control of the immune responses [1]. In particular, the central nervous system may influence immune cell function by the sympathetic nerve fibres within lymphoid organs, which release norepinephrine that can bind to adrenergic receptors expressed by various cell types in the lymphoid organs [2]. The close proximity of sympathetic nerve terminals to immune cells provides a mechanism for the specific targeting of catecholamines to immune cells [3, 4]. On the other hand, little is known about the role of, and the molecular mechanisms underlying, the innervation of the immune system.

Rac GTPases are members of the Rho family, which positively regulate the dynamics of the actin cytoskeleton, thus affecting cell morphology and development [5]. It is known that the endogenous Rac GTPases specifically expressed in each organ play important functions in the development of the neural and immune system respectively [6–8]. Specifically, two of the three mammalian Rac GTPases are expressed in the immune system: the ubiquitous Rac1 and the hematopoietic-specific Rac2 proteins [9, 10]. On the other hand, the neural-specific Rac3/Rac1B GTPase is co-expressed with Rac1 in the nervous system [11, 12], while it is undetectable in the lymphoid organs [13]. We have generated a number of KO mice in which either one or both Rac1 and Rac3 genes have been deleted during neuronal development [14]. These mice include the full deletion of the neural-specific gene for Rac3 (Rac3^KO^), and the conditional neuronal deletion of the gene for Rac1 by the Synapsin-Cre transgene (Rac1^N^). While single Rac3^KO^ or Rac1^N^ mutant mice do not show evident phenotypes and develop normally, Rac1^N^/Rac3^KO^ double mutant mice are neurologically impaired, and die around P13 (postnatal day 13), with identifiable specific defects in the development of the brain [14].

In this study, we have addressed the indirect effects of the deletion of the neuronal Rac proteins on the development of the immune system. We found that the simultaneous inactivation of neuronal Rac1 and Rac3 causes specific defects in the development of the spleen and thymus of the Rac1^N^/Rac3^KO^ double KO mice, and a concomitant defect in the innervation of the thymus of Rac1^N^/Rac3^KO^ mice. Our results show for the first time that the impairment of specific molecular mechanisms underlying neuronal development has striking effects on the development of the immune system, and support the notion of a direct important implication of innervation on the development of the lymphoid organs.

## Results

### Neuronal deletion of Rac1 and Rac3 impacts on lymphoid organ development

**Figure 1:**
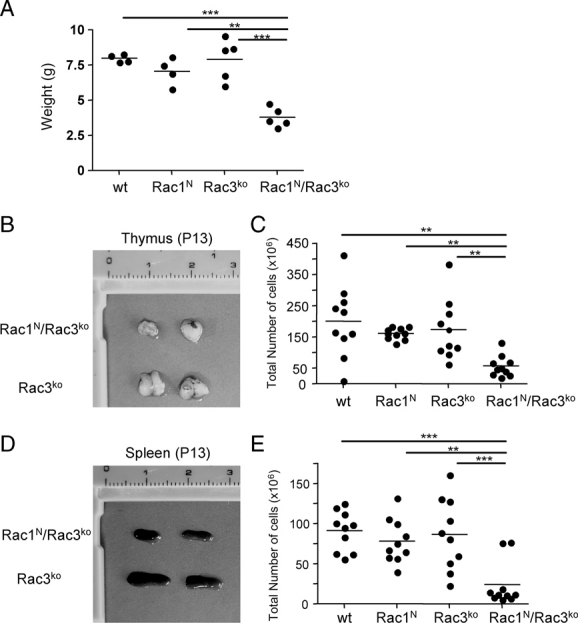
Rac1^N^/Rac3^KO^ double-deficient mice exhibit reduced size and cellullarity of primary and secondary lymphoid organs. WT, Rac1^N^, Rac3^KO^, and Rac1^N^/Rac3^KO^ mice were sacrificed at P13. (A) Body weight of P13 mice. (B and D) Representative (B) thymi and (D) spleens from Rac3^KO^ and Rac1^N^/Rac3^KO^. (C and E) Thymi and spleens were explanted and reduced to single-cell suspensions. Total numbers of viable cells in the (C) thymus and (E) spleen were obtained by (A, C, and E) Trypan blue counts. Each symbol represents data from an individual mouse; horizontal bar represents the mean. ^**^p<0.005; ^***^p<0.001 by one-way ANOVA with Bonferroni correction.

**Figure 2:**
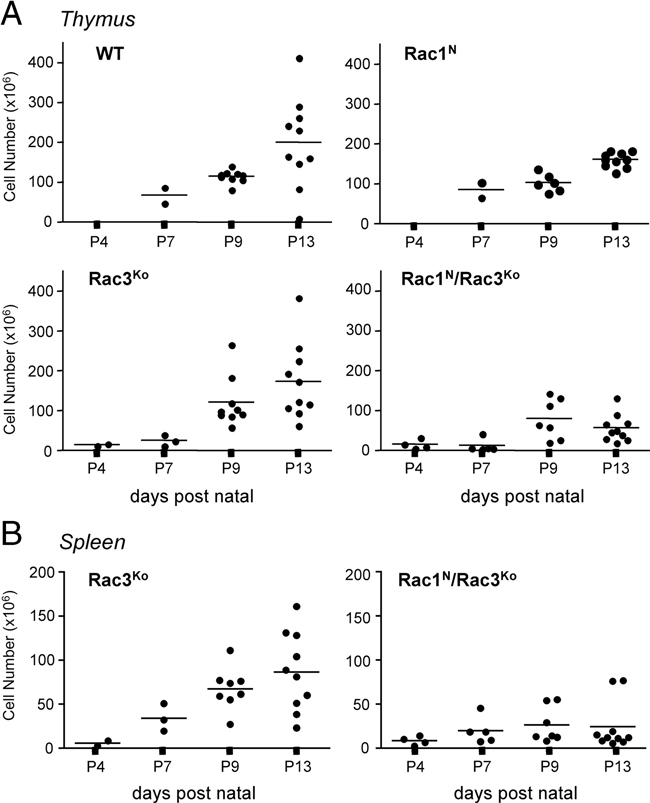
Thymic development declines after P9 in Rac1^N^/Rac3^KO^ mice. (A) WT, Rac1^N^, Rac3^KO^, and Rac1^N^/Rac3^KO^ mice were sacrificed at the indicated postnatal days, and (A) thymocyte and (B) splenocyte numbers were determined by flow cytometry. Total numbers of viable cells are depicted. Each symbol represents data from an individual mouse; horizontal bar represents the mean.

Rac1^N^/Rac3^KO^ double-deficient mice have abnormal postnatal development with severe runting and death by 2 wk of age [14]. By P13 Rac1^N^/Rac3^KO^ double KO mice show clear neurological symptoms [14], are smaller than control littermates (data not shown), and reveal a lower body weight (Fig. 1A). We noticed that by this time, the size (Fig. 1B and D) and the cellularity (Fig. 1C and E) of the thymus and the spleen were severely reduced in the double-mutant mice with respect to Rac3^KO^ littermates (Fig. 1B and D) and to Rac1^N^ and WT mice (data not shown). Accordingly, the total numbers of thymocytes and splenocytes in Rac1^N^/Rac3^KO^ double KO mice were also decreased when compared with WT, Rac1^N^, and Rac3^KO^ littermates (Fig. 1C and E). In contrast, the cellularity of peripheral lymph nodes was comparable in double KO and control littermates (Supporting Information Fig. 1). To better understand this phenomenon we performed longitudinal analyses. Mice were sacrificed at P4, P7, P9, and P13 and organ cellularity was assessed by viable counts. While thymic growth was comparable up to P9 in all the mice and continued to grow in WT, Rac1^N^, and Rac3^KO^ littermates, thymic involution was found by P13 in Rac1^N^/Rac3^KO^ double-mutant mice (Fig. 2A). In the case of the spleen (Fig. 2B), differences were already evident by P9, a time at which both neurological manifestation and reduced body weight are already evident [14].

**Figure 3:**
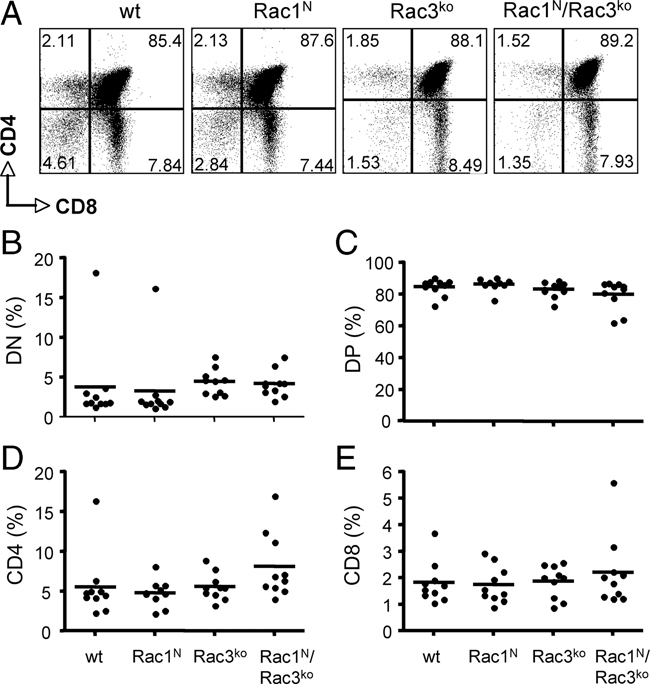
Thymocyte subset composition is preserved in Rac1^N^/Rac3^KO^ double KO mice. WT, Rac1^N^, Rac3^KO^, and Rac1^N^/Rac3^KO^ mice were sacrificed at P13. Thymi were explanted, reduced to single-cell suspensions, and analysed by flow cytometry after staining with anti-CD4, CD8 mAbs. (A) Representative dot plots after gating on viable cells. (B–E) Frequency of (B) CD4/CD8 DN, (C) CD4/CD8 double positive, and (D) CD4 or (E) CD8 single positive (SP) cells of individual mice. Each symbol represents data from an individual mouse; horizontal bar represents the mean. Data were not statistically different by one-way ANOVA with Bonferroni correction.

With the aim of defining the immunophenotype of Rac1^N^/Rac3^KO^ mice, primary and secondary lymphoid organs were examined by flow cytometric analysis using lineage-specific markers during disease progression at P13, a time at which the cellularity of both thymus and spleen is decreased in Rac1^N^/Rac3^KO^ mice. The relative distribution of CD4^−^CD8^−^ double negative (DN) cells and subsets (identified by the relative expression of CD44 and CD25, data not shown), CD4^+^CD8^+^ double positive, and CD4^+^ or CD8^+^ single-positive thymocytes was comparable in WT, Rac1^N^, Rac3^KO^, and double-mutant mice at P13 (Fig. 3). Similar results were obtained at P9 (Supporting Information Table 1). In spite of the fact that the relative representation of thymocyte precursor was mostly preserved, as a consequence of overall decreased cellularity (Fig. 1C and E), the total numbers of all major subsets were reduced in Rac1^N^/Rac3^KO^ double KO mice when compared with WT, Rac1^N^, and Rac3^KO^ littermates (data not shown). This might suggest that thymic defects could not be ascribed to a specific developmental arrest but rather to a general defect of organ function.

**Figure 4:**
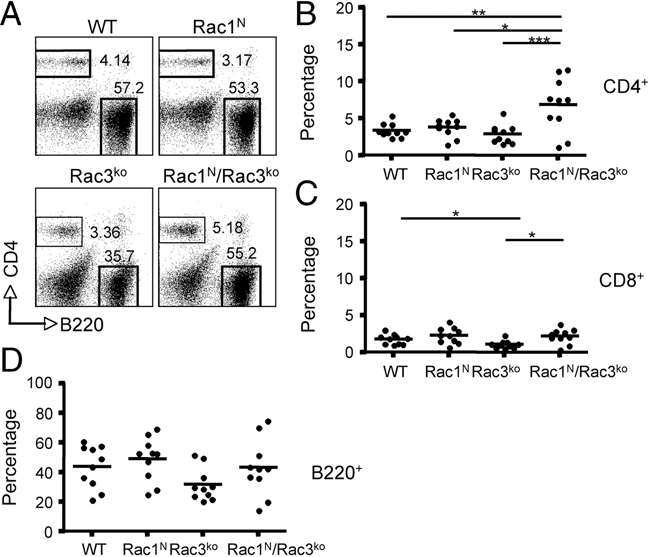
Normal frequencies of mature T and B cells in double KO mice. WT, Rac1^N^, Rac3^KO^, and Rac1^N^/Rac3^KO^ mice were sacrificed at P13. Spleens were explanted, reduced to single-cell suspensions, and analysed by flow cytometry after staining with anti-CD4, CD8 and B220 mAbs. (A) Representative dot plots after gating on viable cells. (B–D) Frequency of (B) CD4-, (C) CD8- and (D) B220-expressing cells expressed as a percentage of total viable cells. Each symbol represents data from an individual mouse; horizontal bar represents the mean. ^*^p<0.05; ^**^p<0.005; ^***^p<0.001 by one-way ANOVA with Bonferroni correction.

At P13, CD4 and CD8 mature T cells were found to be slightly enriched in frequencies in the spleen of double-mutant mice when compared with WT mice, Rac1^N^, and Rac3^KO^ littermates (Fig. 4A–C), although reduced in total number as a consequence of defective cellularity of the organ at this time (Fig. 1E). Also the frequency of mature B220^+^ cells was found to be normal (Fig. 4D), while their total numbers were lower in Rac1^N^/Rac3^KO^ double KO mice when compared with WT, Rac1^N^, and Rac3^KO^ littermates (data not shown). This was in spite of their normal representation in the BM, which revealed comparable frequencies of eritroid, myeloid, and hematopoietic cells in WT and mutant mice (Supporting Information Fig. 2). We next investigated the histological appearance of cryo-preserved P13 tissue sections. The general architecture of the thymus was preserved in Rac1^N^/Rac3^KO^ with proper cortico-medullary differentiation. Immunostaining of stromal components indicated that both mTEC (cytokeratin 5; CK5^+^) and cTEC (CK8^+^) were correctly positioned. Similarly, immunoreactivity for claudin-4, detecting mature mTEC, was comparable (Supporting Information Fig. 3). Immature thymocytes, identified by the TdT, were also found to be correctly positioned and mostly distributed within cortical region with only rare TdT^+^ cells present within the medulla of both Rac3^KO^ and Rac1^N^/Rac3^KO^ (Supporting Information Fig. 3). Although IL-7, a nonredundant trophic factor for immune cell development [15], was expressed in both Rac3^KO^ and Rac1^N^/Rac3^KO^ thymi (Supporting Information Fig. 4A), variability among independent experiments failed to overrule a possible defect along this pathway in mutant mice. Against this possibility, DN thymocytes proliferated to comparable extents in short BrdU-pulse experiments in vivo (Supporting Information Fig. 4C). In some of the Rac1^N^/Rac3^KO^ sections the cortical region appeared to contain fewer CD3^+^ precursors, which instead appeared better represented within the more densely populated medullary region. Also, Foxp3^+^ cells, identifying T-regulatory cells exclusively present in the thymic medulla, were better represented in Rac1^N^/Rac3^KO^ when compared with Rac3^KO^ mice (Supporting Information Fig. 3). Histological analysis of the spleen revealed an altered red/white pulp ratio in Rac1^N^/Rac3^KO^, possibly reflecting the lower numbers of T and B cells, major constituents of the white pulp (Supporting Information Fig. 5A and B).

In addition to the thymus and the spleen, the size (data not shown) and weight (Supporting Information Fig. 6A) of other organs including kidney, heart, and liver were also significantly smaller in P13 double KO animals when compared with Rac3^KO^ littermates. In contrast, the size and weight of the brain [14] and the lung (Supporting Information Fig. 6A) were comparable in Rac3^KO^ and Rac1^N^/Rac3^KO^ mice. In all cases, the histological appearance of non-lymphoid tissues of Rac1^N^/Rac3^KO^ mice appeared normal (Supporting Information Fig. 6B).

Together these data indicate that the manifestation of neurological symptoms influences the development/growth of several vital organs to different extents, severely hindering immune-competence in Rac1^N^/Rac3^KO^ double-mutant mice.

### Neuronal depletion of Rac1 and Rac3 is responsible for the effects observed on lymphoid organs

**Figure 5:**
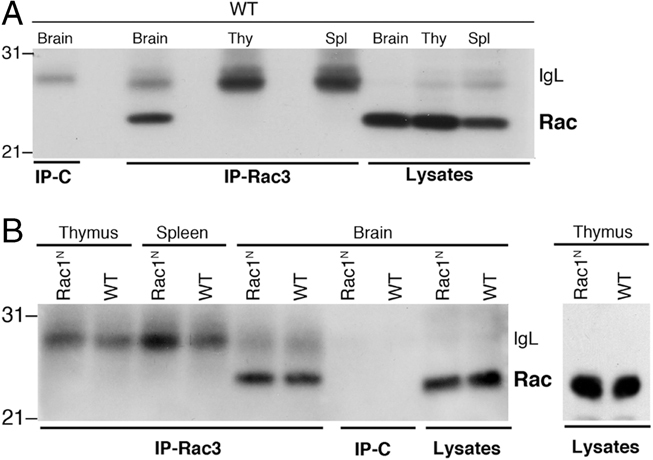
Rac3 is not expressed in lymphoid tissues of WT and Rac1^N^ mice. WT and Rac1^N^ mice were sacrificed at P7. Protein lysates of brain, thymus (Thy), and spleen (Spl) from (A and B) WT and (B) Rac1^N^ mice were immunoprecipitated with control (IP-C) or anti-Rac3 antibodies (IP-Rac3). Immunoprecipitates and lysates (200 μg) were blotted with an anti-Rac mAb recognizing both Rac1 and Rac3. Numbers on the left indicate molecular weight. Data are representative of three independent determinations.

Rac1^N^/Rac3^KO^ mice carry the full deletion of the neural-specific gene for Rac3 (Rac3^KO^), and the conditional neuronal deletion of the gene for Rac1 by the Synapsin-Cre transgene (Rac1^N^). We reasoned that the developmental defects observed in the spleen and thymus of Rac1^N^/Rac3^KO^ mutant mice was not attributable to a defect of endogenous Rac function in these organs. To better analyse this possibility we investigated whether Rac was expressed in the thymus and spleen by immunoblots. While Rac3 was readily immunoprecipitable from brain extracts, it remained undetectable in thymus and spleen extracts of both WT and Rac1^N^ mice (Fig. 5A and B). Furthermore, Rac levels were unchanged in the thymus of Rac1^N^ with respect to WT mice (Fig. 5B), supporting preserved Rac representation in the lymphoid compartment of Rac1^N^/Rac3^KO^ mutant mice. Thus, the finding that Rac3 is not expressed in the thymus and spleen, together with the observation that lymphoid organ aplasia was only found after full deletion of Rac3 and neuronal deletion of Rac1 strengthens the notion that rather than a defect intrinsic to the lymphoid tissues, an extrinsic mechanism was responsible for the observed impairment of the immunocompetence.

**Figure 6:**
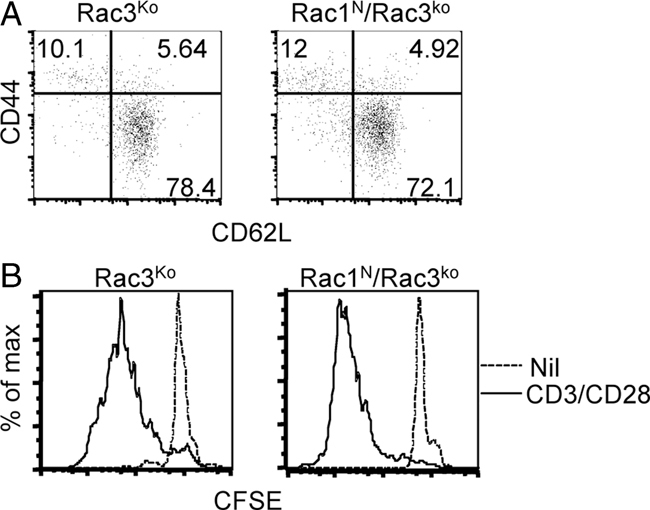
Mature T cells from double KO mice reveal a naïve phenotype and normal proliferative capacities. (A) Rac3^KO^ and Rac1^N^/Rac3^KO^ mice were sacrificed at P13. Splenocytes were recovered and analysed by flow cytometry following staining with anti-CD4, CD44, and CD62L mAbs. The plots are gated on viable CD4^+^ T cells. Data from one representative mouse out of ten analysed are depicted. The frequency of naïve (CD44^low^, CD62L^high^), central memory (CD44^high^, CD62L^high^), and effector (CD44^high^, CD62L^low^) cells is depicted. (B) Splenocytes were labelled with CFSE and cultured in the absence (Nil) or presence of CD3/CD28 mAbs. After 5 days cells were stained with anti-CD4 and CD8 mAb and analysed by flow cytometry. Representative histograms depict viable CD4^+^ cells. Similar results were obtained for CD8^+^ cells (data not shown).

To further understand whether neuronal depletion of Rac1 and Rac3 had an impact on mature T-cell function, we characterized their surface and functional phenotype. In spite of the observed peripheral lymphopenia, central (CD44^high^CD62L^high^) and effector (CD44^high^CD62L^low^) memory cells were equally represented among CD4^+^ (Fig. 6A) and CD8^+^ (data not shown) T cells derived from Rac1^N^/Rac3^KO^ and Rac3^KO^ littermates. Most T lymphocytes maintained a naïve CD44^low^CD62L^high^ phenotype (Fig. 5A), proper expression of CD127 (Supporting Information Fig. 4B), and a low rate of cell division in short BrdU-pulse experiments in vivo (Supporting Information Fig. 4D). Furthermore, flow cytometry CFSE dilution assays revealed that CD4^+^ (Fig. 6B) and CD8^+^ (data not shown) T cells from Rac1^N^/Rac3^KO^ and Rac3^KO^ littermates proliferated to a similar extent after polyclonal CD3/CD28-driven stimulation in vitro. These results support the existence of extrinsic rather than cell autonomous defects.

### Axonal thymic innervation is suboptimal in Rac1^N^/Rac3^KO^ double KO mice at P13

**Figure 7:**
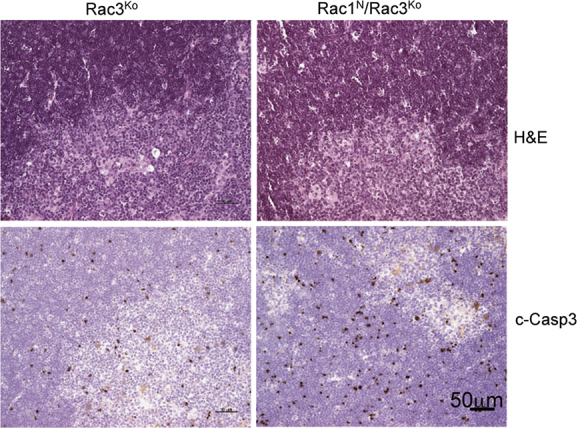
Thymic involution in double KO mice reflects increased apoptosis. Rac3^KO^ and Rac1^N^/Rac3^KO^ were sacrificed at P13. Cryosections from the thymus were analysed by hematoxylin and eosin staining, and immunohistochemistry with anti-cleaved caspase 3 (c-Casp3) antibody. Bar: 50 μm.

**Figure 8:**
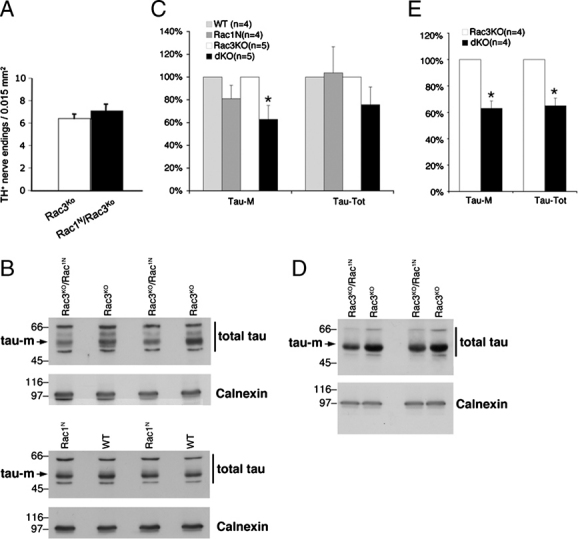
Suboptimal thymic innervation in Rac1^N^/Rac3^KO^ double KO mice. (A) Cryosections of the thymus from P13 Rac1^N^/Rac3^KO^ and control Rac3^KO^ littermates were stained with anti-TH antibodies and analysed by confocal microscopy (representative images are shown in Supporting Information Fig. 7). The density of TH^+^ fibres was quantified and expressed as the number of TH^+^ fibres per 0.015 mm^2^ of thymic cortex. Data are mean±SEM of 80 fields from eight thymic sections per genotype from three independent experiments. Student *t* test: *p*=0.24 comparing control Rac3^KO^ and Rac1^N^/Rac3^KO^ littermates. (B–E) Tau and calnexin expression was evaluated by immunoblotting thymic lysates (100 μg protein per lane; for each comparison, two distinct pairs of littermates are shown) obtained at (B and C) P13 and (D and E) P9. (C and E) Expression of the major intermediate band of tau (tau-m) and of total tau (equal to the sum of the signals for all the bands specifically recognized by the anti-tau antibody) was normalized to that of calnexin and presented as mean±SEM (*n*=4–5 different thymi per mouse genotype). ^*^p<0.05 by the Student's *t* test comparing control Rac3^KO^ and Rac1^N^/Rac3^KO^ littermates.

Progressive loss in cellularity in the thymus and the spleen of Rac1^N^/Rac3^KO^ mice was accompanied by an increased rate of apoptosis, revealed by immunohistochemistry with anti-cleaved caspase 3 mAb, both in the thymus cortex and medulla (Fig. 7), and in the spleen (Supporting Information Fig. 5C). As progressive loss of immunocompetence parallels the onset of neurological manifestations, we investigated whether defective organ innervation might account for the observed phenotype. Innervation of the thymus by non-myelinated sympathetic and parasympathetic nerves has been shown to be important for thymus function and to maintain T-cell maturation. We analysed the expression in the thymus of tyrosine hydroxylase (TH), a key enzyme in the synthesis of norepinephrine, which has been used previously to study axonal endings from sympathetic motor neurons in the thymic cortex, and associated with thymic involution in Twitcher mice [16]. We found that at P13, the density of sympathetic innervation of the thymus was comparable in Rac1^N^/Rac3^KO^ double mutants and control Rac3^KO^ mutants, indicating that a defect at this level might not account for the observed phenotype (Fig. 8A and Supporting Information Fig. 7). We also studied cholinergic innervation by immunohistochemistry. Unfortunately, while antibodies to choline acetyltransferase, a marker of cholinergic innervation, revealed specific signals on control brain sections, they failed to provide a reliable signal on thymic sections (data not shown).

In the case of neurofilament, expression in the thymus of double KO mice was comparable to that of control tissue (Supporting Information Fig. 8). Antibodies for other neuronal markers also failed to provide a specific signal or revealed high non-specific background on lymphoid tissue sections (Supporting Information Fig. 9A and data not shown). In the case of the Tuj1 mAb for β3 tubulin it has been found to be also expressed in lymphoid cells [17], and only detectable in neuronal terminals in immunologically mature mice by confocal microscopy [18]. We thus attempted to address the quantitative expression of neuronal markers in the thymus by immunoblotting of tissue extracts. Quantification of β3 tubulin did not show any significant differences between the thymi of double KO and control mice (Supporting Information Fig. 9B). On the other hand, we found that the microtubule-associated protein tau, a specific axonal marker [19], was detectable in the thymus of P13 mice (Fig. 8B). Specifically, we observed a significant reduction (≈40%) of the major intermediate band (tau-m) recognized by the anti-tau antibody in the thymus of Rac1^N^/Rac3^KO^ double KO mice with respect to control Rac3^KO^ littermates (Fig. 8C). No significant differences were detected between Rac1^N^ and WT P13 littermates when quantified in independent experiments (Fig. 8B and C). The decrease of tau in the thymi of double KO mice with respect to control Rac3^KO^ mice was evident already at P9 (Fig. 8D and E). No differences were detectable for either tau or β3 tubulin in the spleen from P13 double KO mice with respect to controls (Supporting Information Fig. 10). These results indicate that axonal endings within the thymus are compromised in Rac1^N^/Rac3^KO^ double-mutant mice, providing a possible causative link between neuronal perturbation and the observed thymic deficits.

## Discussion

The results from this study show that the specific neuronal deletion of the genes for the two GTPases Rac1 and Rac3 hinders the acquisition of immune-competence, and this can be explained, at least in part, by defective thymus innervations by tau-positive neurons.

Rac1^N^/Rac3^KO^ double-deficient mice die by 2 wk of age [14]. By P13 Rac1^N^/Rac3^KO^ double KO mice show clear neurological symptoms [14] and reduced body weight when compared with control littermates. While brain, lungs, and lymph nodes of Rac1^N^/Rac3^KO^ double KO were comparable to those of Rac3^KO^ mice, kidney, heart, liver, thymus, and the spleen were found to be smaller in size. Behavioural analysis of the mice (observation) indicated the absence of nutritional problems. Furthermore, histological examination of tissue sections revealed normal for most of the organs, except for the spleen, suggesting that the loss of immune-competence is not the result of a general metabolic stress. Results rather indicate that neurological manifestations might impact the function of several body organs to different extents, possibly with different kinetics and mechanisms, to which the lymphoid organs might be most sensitive.

In longitudinal analysis we observed that the thymus and the spleen progressively enlarged during postnatal life, doubling in size between P4 and P13 in normal WT mice, in mice lacking Rac3, or in mice with neuronal specific inactivation of Rac1. In contrast, in Rac1^N^/Rac3^KO^ double-mutant mice thymic growth was normal up to P9 but severely hindered at later times. In the case of the spleen, but not the peripheral lymph nodes, differences between Rac1^N^/Rac3^KO^ and control littermates were evident even at P9. Of note, in addition to T cells, also B cells were reduced in numbers in Rac1^N^/Rac3^KO^ mice. This was in spite of proper representation of BM precursors, and rather reflected a peripheral defect evidenced by the disrupted architecture of the spleen.

Reduced thymic output, together with hindered homeostasis of mature T (and B) cells caused a profound peripheral lymphopenia in the double KO animals. Of note, although cells of maternal origin might contribute to the circulating pool of mature T cells, their frequency in offspring is generally very low (<0.1%) [20]. Since double-mutant mice are indistinguishable from controls up to P7, it seems unlikely that maternal lymphocytes account for all circulating cells. In spite of the lymphopenic environment, most of the CD4^+^ and CD8^+^ T cells in the periphery maintained a naïve phenotype (CD44^low^, CD62L^high^, CD127^high^), and a very low frequency of cells incorporated BrdU in short pulse experiments in both double KO and control littermates. These findings suggest that proliferation of mature T-cell precursors, commonly observed in lymphopenic conditions and known to cause naïve T cells to acquire a memory phenotype, does not play a relevant role by this early age of postnatal life. Thus, the lower T-cell numbers observed in double KO mice have to be ascribed to extrinsic mechanisms rather than to defects intrinsic to the lymphocytes. Accordingly, (i) T cells do not express Rac3, but rely on Rac1 and Rac2 [9, 10] that were not affected in the lymphoid organs of Rac1^N^/Rac3^KO^ mice; (ii) T cells derived from Rac1^N^/Rac3^KO^ double-mutant mice were able to respond to TCR/CD28 stimulation, and to proliferate to extents comparable to those of WT cells, supporting a preserved functionality. Accordingly, previous reports indicated that deletion of the Rac1 and Rac2 genes within the lymphoid cells evoked immune phenotypes that differed from those described here. For instance, Rac2-deficient mice revealed normal thymocyte development or positive/negative selection, and only impaired actin polymerization, Ca2^+^ generation, and Erk and p38 activation during T-cell stimulation and Th1 differentiation [21]. Furthermore, mice with hematopoietic-specific inactivation of Rac2 developed normally with weight and cellularity of spleens and thymi similar to those of control mice, and only revealed defective chemotaxis and superoxide production by neutrophils [22]. Whereas the absence of either Rac1 or Rac2 alone had no effect on thymic development, their simultaneous deletion resulted in a developmental arrest, defective positive selection, and enrichment of CD4^–^CD8^–^ DN thymocytes [23, 24]. B cell-lineage-specific inactivation of Rac1 in a Rac2-deficient background [25] showed no significant changes in the number of pro-, pre-, and immature B cells, but profound defects in the number of splenic transitional types 1 and 2 and mature B cells [26]. Thus, while lymphoid-specific inactivation of Rac family members appears to interfere with proper central development and peripheral mature lymphocyte functions, our data rather indicate that the effects observed on the immune system are due to the ablation of Rac GTPases in the neuronal cells. Indeed, in the case of Rac1^N^/Rac3^KO^ mice, the neuronal deletion of Rac1 and Rac3 did not cause a specific arrest at selected stages during thymic development and did not influence the ability of T lymphocytes to proliferate upon receptor engagement, thus supporting the lack of a T-cell intrinsic defect and rather the existence of a neuronal deficiency, impacting on organ innervation. Indeed the thymus of double KO mice revealed a decreased level of the microtubule-stabilizing protein tau, a specific axonal marker [19]. Whether decreased innervation by tau^+^neurons might also explain the reduction in size of the spleen and eventually of the kidney, heart, and liver with consequences that might become histologically visible at different times remains to be determined.

The mechanisms that underlie the possible influence of neuronal function on the development of the immune system are poorly understood. Previous results suggested that the thymus receives parasympathetic innervations relatively early in ontogeny, and that nerve fibres could be involved in the regulation of the organ activity through action upon the thymocytes and/or by modulation of the thymic vasculature or epithelial cell activity [27]. Direct innervation [1, 28, 29] as well as the release of hormones such as somatotrophin [30] have also been previously shown to exert an influence on primary and secondary immune organs, which are highly innervated by non-myelinated sympathetic projections from central motor neurons [31, 32]. We have shown here that perturbation of the function of neuronal Rac GTPases during neuronal development has a profound effect on the development of a functional immune system. To the best of our knowledge, this is the first indication that perturbation of the machinery directly involved in neurite extension affects the development of the immune system. Moreover, our finding that the thymus of double Rac1/Rac3 mice showed decreased expression of the axonal protein tau provides support to a neuronal contribution to the homeostasis of primary lymphoid organs. In this direction, it has recently been shown that the degeneration of the autonomic innervation observed in the Twitcher mouse model for Krabbe disease, a neurological disorder characterized by progressive demyelination, is also associated to thymic atrophy and loss of immune-competence [16], supporting a crucial role for neuronal function in the regulation of the functions of lymphoid organs. Progressive involution of the thymus starting at P9 in Rac1^N^/Rac3^KO^ double-mutant mice closely resembles that found after P15 in the Twitcher mouse. As in Rac1^N^/Rac3^KO^ double-mutant mice, Twitcher mutants also revealed a normal development of thymi and peripheral lymphoid organs during the initial postnatal life. Thus, progressive loss of immune-competence, best explained by thymic involution, found at P13 and P30 characterize both the Rac1N/Rac3^KO^ and Twitcher mice, respectively, which substantially differ in causes and manifestation of neurological dysfunctions but recapitulate similar immunological phenotypes. Of note, while there is a predominate sympathetic (catecholamine) input to all components of the immune system, afferent innervation might be limited to lymph nodes and BM. Thus, differences in organ innervations, in the relative contributions of Rac1 and Rac3 to the development of nerve fibres serving distinct organs, or in innervation of the organ-associated vasculature, might account for the fact that functionality of spleen and thymus but not of BM or lymph nodes are impeded during initial postnatal life. Similarly, the finding that both the thymus and spleen mainly benefit from sympathetic nerves, which, however, arise from different ganglia (from the upper paravertebral ganglia of the sympathetic chain and the superior mesenteric and celiac ganglionic plexuses respectively [33]), might explain the difference in the timing of symptom manifestation.

Hypoplasia and abnormal maturation of lymphoid organs have been documented in other diseases, including the wasted mouse and the MND2 mouse models of motor neuron disease [34], or the staggered mouse [35], and after experimental axonal loss [36–38]. Our data support this notion and further indicate that neuronal defects impede immune-competence regardless of the time of occurrence. Given the crucial role for neuronal function in the regulation of central and peripheral competence, it would be important to define appropriate monitoring and therapeutic options for patients with neurological dysfunctions likely at risk for immune-incompetence development.

## Materials and methods

### Mice

Animal care was in accordance with institutional guidelines. Procedures for the generation of Rac1^N^, Rac3^KO^, and Rac1^N^/Rac3^KO^ mice, and for their genotyping have been described [14]. For experiments, Rac1^F/F^ mice were crossed with Rac1^F/+^SynI-Cre mice to obtain Rac1^F/F^ and Rac1^F/F^SynI-Cre littermates, defined as WT and Rac1^N^ respectively; whereas Rac1^F/F^Rac3^KO^ mice were crossed with Rac1^F/+^SynI-Cre/Rac3^KO^ mice to obtain Rac1^F/F^/Rac3^KO^ and Rac1^F/F^SynICre/Rac3^KO^ littermates, defined as Rac3^KO^ and Rac1^N^/Rac3^KO^ respectively.

### Antibodies

The following antibodies were used for biochemical and morphological analysis: anti-calnexin polyclonal antibody (pAb) (Sigma); anti-tau mAb, anti-choline acetyltransferase (Chemicon International, Temecula, CA); anti-β3 tubulin (Tuj1, from Berkeley Antibody Company, Richmond, CA); anti-neurofilament (Affinity); anti-TH (from Neuromics); anti-Rac3 pAb [13]; anti-Rac mAb (Upstate Biotechnology, Lake Placid, NY); anti-active caspase-3 mAb (BD Biosciences, San Jose, CA); anti-CD3ɛ (clone CT-CD3; Valter Occhiena); anti-cytokeratin 5 (Covance); anti-cytokeratin 8 and anti-claudin-4 (Zymed), anti-FoxP3 (eBioscience), anti-TdT (DAKO).

### Flow cytometry analysis

Mice were sacrificed at P4–P13. Thymus, peripheral lymph nodes, and spleen were collected, and homogenized into single-cell suspensions. Viable cells were enumerated by Trypan Blue-assisted counts. The cells were then incubated with a blocking buffer (5% rat serum and 95% culture supernatant of 2.4G2 anti-FcR mAb-producing hybridoma cells) for 20 min at 4°C to saturate the FcRs. Cells were then stained for CD4, CD8, B220, CD44, CD62L mAb (BD Biosciences). Thymocyte subpopulations were detected by staining with mAbs for CD4, CD8, CD25, and CD44. Samples were collected on a FACSCANTO (Becton Dickinson) and then analysed using Flow Jo software.

When indicated cells were labelled with the vital dye CFSE as described previously [39], stimulated with plate-bound anti-CD3 (2C11) and anti-CD28 (37.51) mAbs, and analyzed by flow cytometry.

### Histology and immunohistochemistry

Organs isolated from P13 mice were cryosectioned and processed for diaminobenzidine light or fluorescence confocal immunocytochemistry as described previously [13, 40]. Sections were were stained with the indicated antibodies and counterstained with DAPI. Hematoxylin staining was performed on cryosections with Hematoxylin QS (Vector Labs). Sections were observed through an Ultraview (PerkinElmer) and a Zeiss upright microscope with Nomarski optics.

### Biochemical analysis

Organs isolated from P9 or P13 mice were lysed with nine volumes of lysis buffer (0.5–1% Triton X-100, 150 mM NaCl, 20 mM Tris-Cl, pH 7.5, 1% sodium fluoride, 1% sodium orthovanadate, cocktail of protease inhibitors). Cleared lysates were used for immunoblotting and immunoprecipitation with the indicated antibodies. For the detection of Rac3 in organs, lysates were incubated with anti-Rac3 antibody pre-adsorbed to protein A Sepharose beads, washed, and used for analysis by SDS-PAGE and immunoblotting with anti-Rac mAb recognizing all Rac proteins (Upstate Biotechnology). The first antibodies were revealed either by ^125^I-protein A (Amersham) or by HRP-conjugated anti-rabbit or anti-mouse immunoglobulins (ECL, Amersham). For the quantification of the protein levels, bands from the immunoblots were analysed with a Personal SI Laser Densitometer (Molecular Dynamics, Sunnyvale, CA).

### Statistical analysis

Data were analysed by the Student's *t* test or by one-way ANOVA with Bonferroni correction: ^*^p<0.05; ^**^p<0.005; ^***^p<0.001 comparing single or double KO with WT mice. Statistically significant differences are indicated in the figures.

## Abbreviations

DN: double negative
GTP: guanasine triphosphate
MW: molecular weight
P: postnatal day
pAb: polyclonal antibody
TEC: thymic epithelial cells
TH: tyrosine hydroxylase
