# Supplementary material for: Absence of Rac1 and Rac3 GTPases in the nervous system hinders thymic, splenic and immune-competence development
Source: Eur J Immunol. 2011 Mar 7;41(5):1410–9. doi: 10.1002/eji.201040892 (PMC3132589; doi:10.1002/eji.201040892)
Supplement: Supplementary file 1 [file eji0041-1410-SD1.pdf]

European Journal of Immunology

Supporting Information

for

DOI 10.1002/eji.201040892

Absence of Rac1 and Rac3 GTPases in the nervous system hinders thymic, splenic and immune-competence development

Veronica Basso, Sara Corbetta, Sara Gualdoni, Diletta Tonoli, Pietro Luigi Poliani, Francesca Sanvito, Claudio Doglioni, Anna Mondino and Ivan de Curtis

© 2011 WILEY-VCH Verlag GmbH & Co. KGaA, Weinheim

Table 1. Frequency of thymocyte subsets in developing thymuses			
**DN (% ± SD)**			
	**p4**	**p7**	**p9**
**wt**		3.92±0.89	3.49±0.67
**Rac1^{N}**		3.40±0.06	3.11±0.59
**Rac3^{KO}**	6.07±0.39	5.91±0.82	4.01±0.43
**Rac1^{N} /Rac3^{KO}**	7.74±3.27	11.83±4.69**	4.25±0.89
**DP (% ± SD)**			
**wt**		87.05±1.48	86.92 ±1.43
**Rac1^{N}**		86.65±0.21	86.37 ±1.09
**Rac3**	82.7±0.57	83.50±1.93	86.60 ±1.58
**dKO**	77.6±8.67	76.74±6.39**	85.56 ±2.16
**CD4SP (% ± SD)**			
**wt**		3,79 ±0.19	4.75 ±1.03
**Rac1^{N}**		3.78 ±0.04	5.39 ±0.74
**Rac3^{KO}**	6.24±0.43	3.71 ±1.61	4.43 ±0.61
**Rac1^{N} /Rac3^{KO}**	7.61±2.96	2.21 ±1.46	5.33 ±0.64
**CD8SP (% ± SD)**			
**wt**		1.57 ±0.18	2.29 ±0.85
**Rac1^{N}**		2.37 ±0.38	2.6 ±0.59
**Rac3^{KO}**	2.28±0.08	2.11 ±0.25	1.88 ±0.37
**Rac1^{N} /Rac3^{KO}**	3.66±1.84	2.33 ±0.66	1.78 ±0.33

Supporting Information Table 1. Frequency of thymocyte subsets in developing thymuses. WT, Rac1^{N}, Rac3^{KO}, and Rac1^{N}/Rac3^{KO} mice were sacrificed at the indicated postnatal days. Thymi were explanted, reduced to single cell suspensions, and analyzed by flow cytometry after staining with anti-CD4, CD8 mAbs. Frequency of CD4/CD8 double negative (DN), CD4/CD8 double positive (DP), and CD4 or CD8 single positive (SP) cells of individual mice are depicted. ** $P < 0.005$ by the Student t test comparing wt and Rac1^{N}/Rac3^{KO} mice.

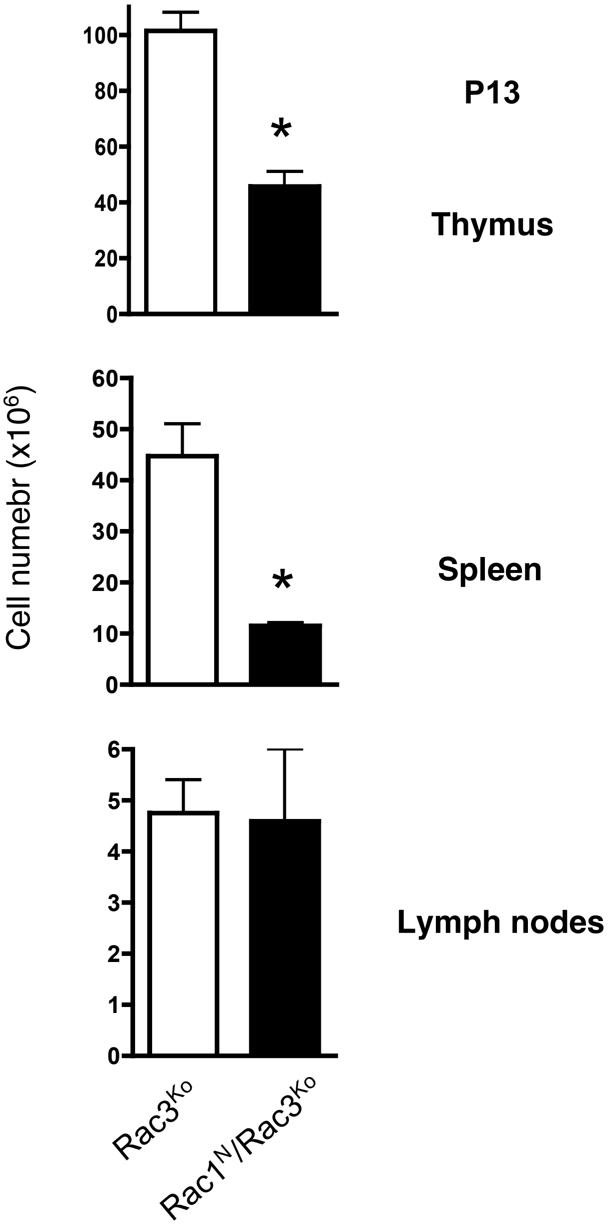

Supporting Information Figure 1. Lymph nodes cellularity is preserved in P13 Rac1^{N}/Rac3^{KO} double deficient mice. Rac1^{N}/Rac3^{KO} and Rac3 ^{KO} control littermates were sacrificed at P13. Thymi, spleens and peripheral lymph nodes (axillary, brachial and cervical) were explanted and reduced to single cell suspensions. Total numbers of viable cells obtained by Trypan blue counts is depicted. * $P < 0.05$ by the Student *t* test.

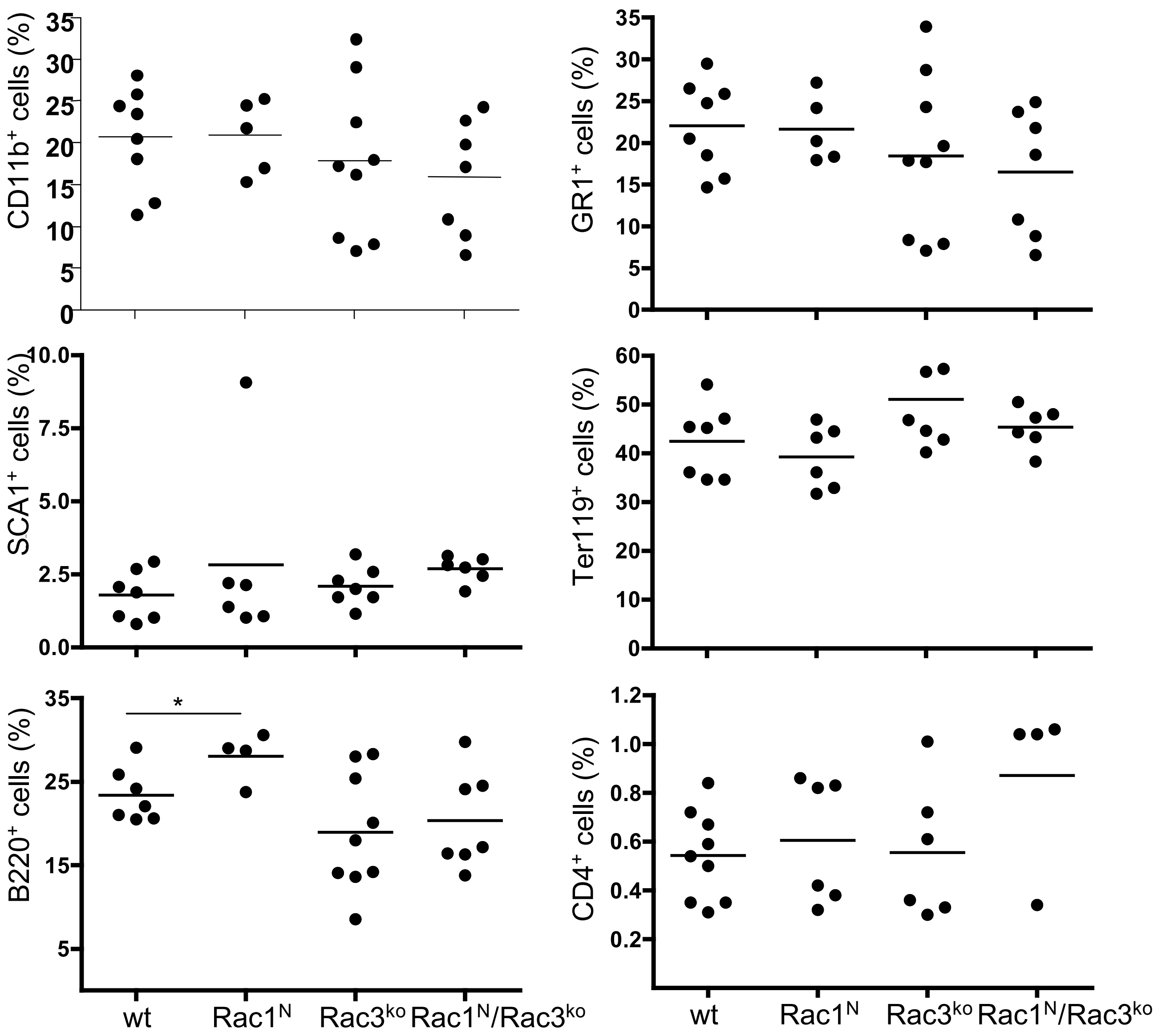

Supporting Information Figure 2. Analysis of bone marrow. WT, $Rac1^{N}$, $Rac3^{KO}$, and $Rac1^{N}/Rac3^{KO}$ mice were sacrificed at P13. Bone marrow was obtained from femur and tibia by flushing. Cell suspensions were analyzed by flow cytometry after staining with anti-CD11b, anti-GR-1, anti-SCA-1, anti-Ter119, anti-B220 and anti-CD4 mAb.

A

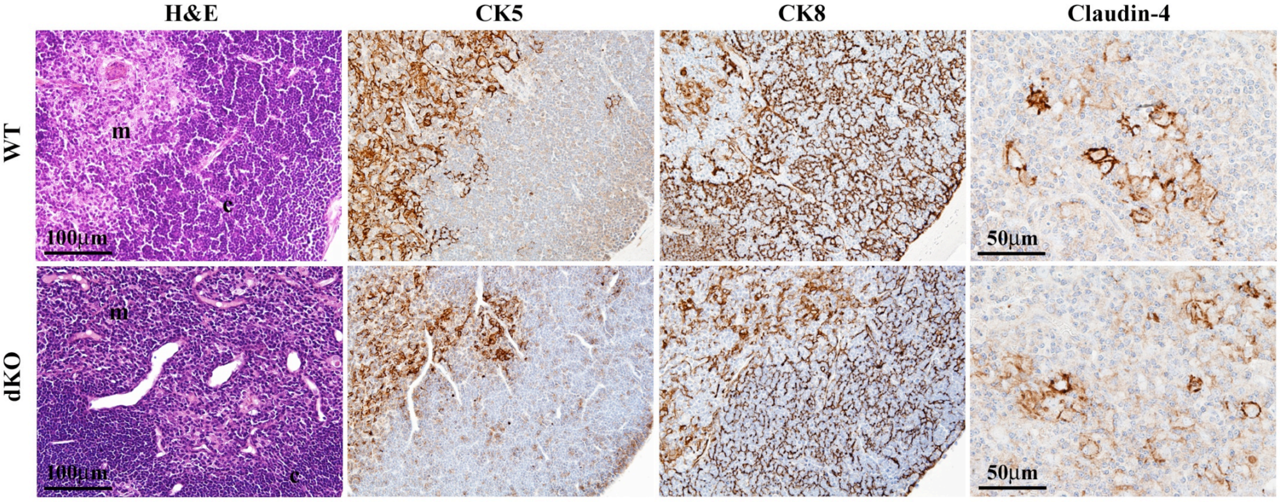

B

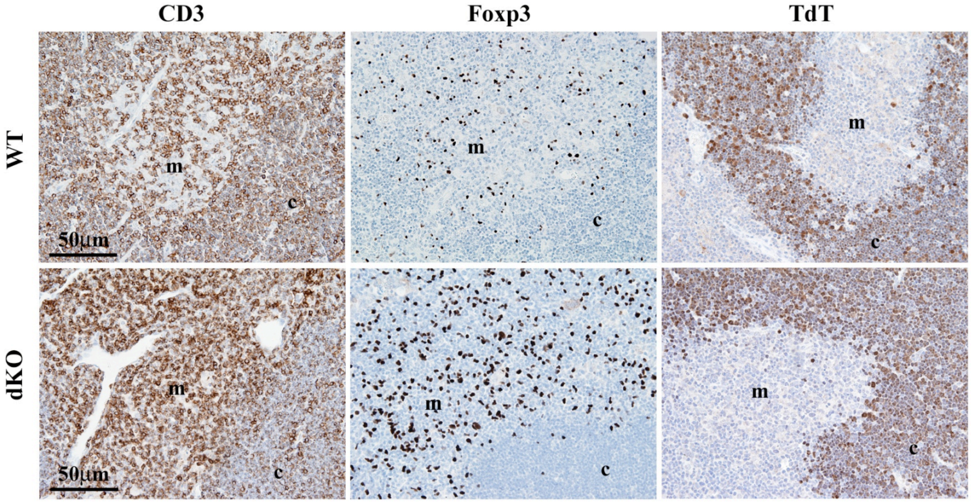

Supporting Information Figure 3. Histological analysis of the thymus of P13 Rac3^{KO} and Rac1^{N}/Rac3^{KO}. Cryosections of thymus derived from P13 Rac3^{KO} and Rac1^{N}/Rac3^{KO} animals were analyzed by hematoxylin and eosin staining (H&E) and by immunohistochemistry with anti-cytokeratin 5 (CK5), anti-cytokeratin 9 (CK9), anti-claudin-4, anti-CD3, anti-Terminal deoxynucleotidyl Transferase (TdT) and anti-FoxP3 mAb. A) Bar: 100µm, with the exception of Claudin-4 (50µm) and B) 50µm. m: medulla; c: cortex.

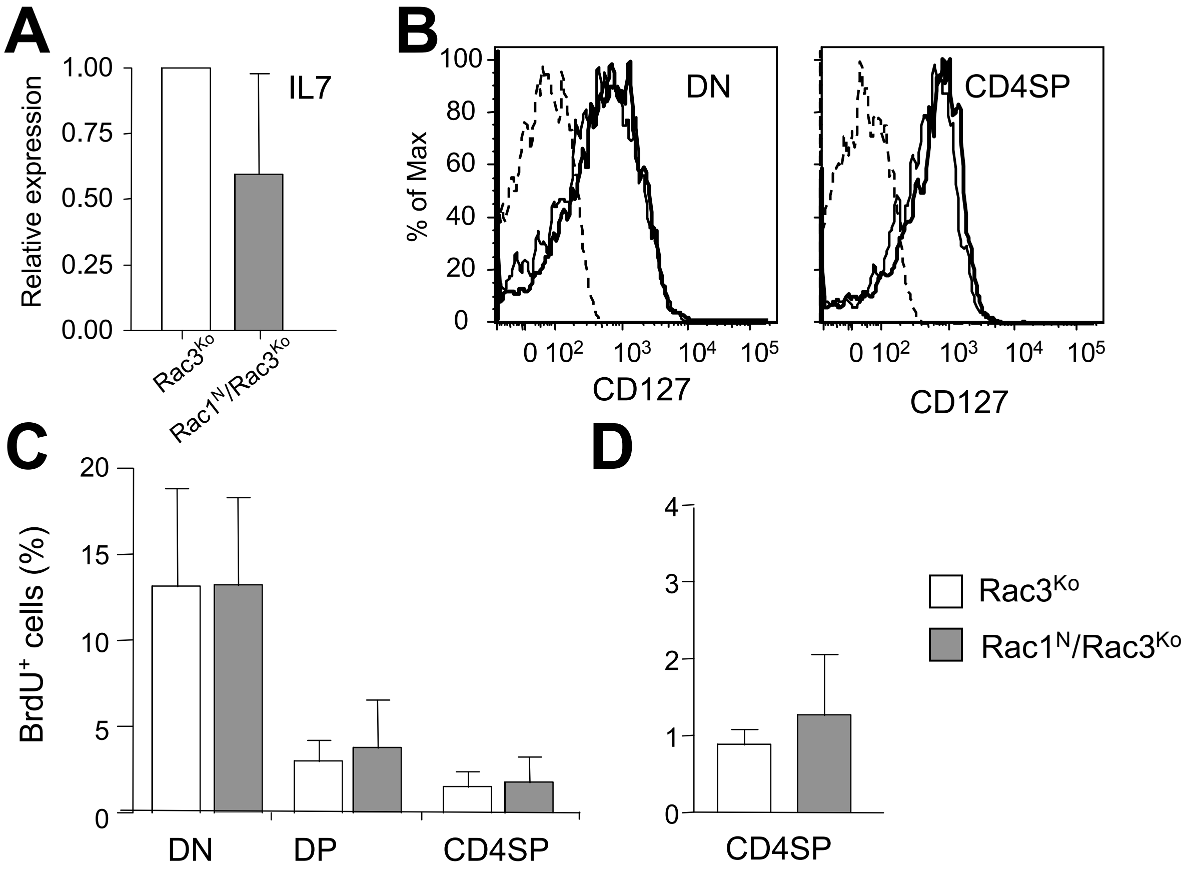

Supporting Information Figure 4. Homeostasis of thymocytes and mature lymphocytes in P13 Rac3^{KO} and Rac1^{N}/Rac3^{KO} mice. Thymi were explanted and analyzed by realtime PCR. Data obtained from several mice from separate litters were normalized to the housekeeping gene TBP and expressed relative to Rac3^{KO} controls. **B**) CD127 surface level was analyzed by flow cytometry after staining for CD4 and CD8. Histograms are shown after gating on DN, DP and CD4SP events (dotted line: control; thin line Rac3^{KO}; thick line: Rac1^{N}/Rac3^{KO}). **C-D**) Twelve days old Rac3^{KO} and Rac1^{N}/Rac3^{KO} mice were given an i.v. injection of Bromodeoxyuridine (5-bromo-2-deoxyuridine, BrdU) and sacrificed 24 hours later. Thymi and spleens were reduced to single cell suspension and analyzed by flow cytometry after staining with anti-CD4, anti-CD8 and anti-Bromodeoxyuridine (or isotype control). The frequency of BrdU^{+} cells of the indicated populations identified by electronic gating is shown.

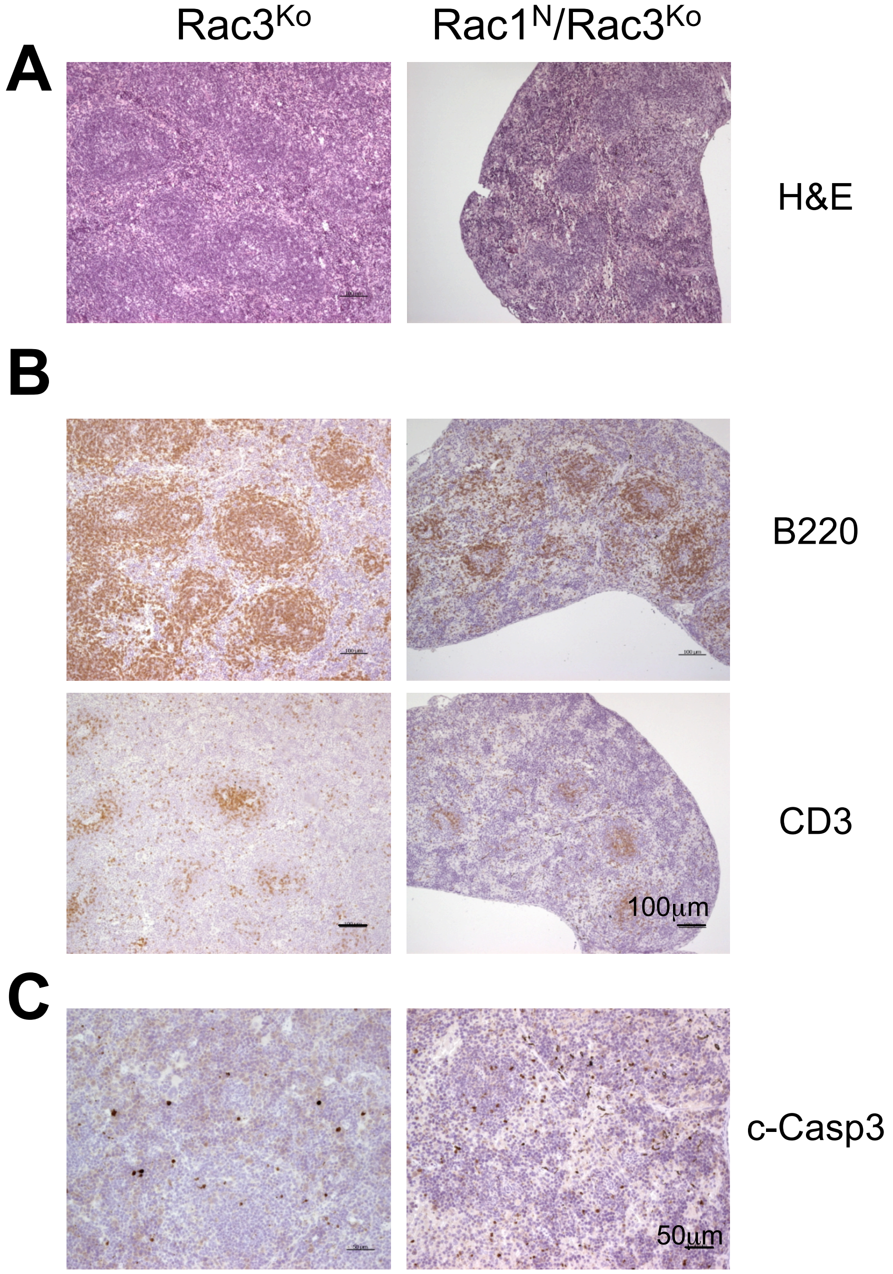

Supporting Information Figure 5. Histological analysis of the spleen of P13 Rac3^{KO} and Rac1^{N}/Rac3^{KO}. Cryosections of spleens derived from P13 Rac3^{KO} and Rac1^{N}/Rac3^{KO} animals were analyzed by hematoxylin and eosin staining (H&E) (**A**) and by immunohistochemistry with anti-B220, CD3 (**B**) and cleaved caspase 3 (c-Casp3) (**C**) antibodies. A lower organization of the white pulp, paralleled by a higher frequency of cleaved caspase 3-positive (c-Casp3) cells is evident in the spleen of Rac1^{N}/Rac3^{KO} when compared to Rac3^{KO} mice. Bar: (**A, B**) 100μm, (**C**) 50μm.

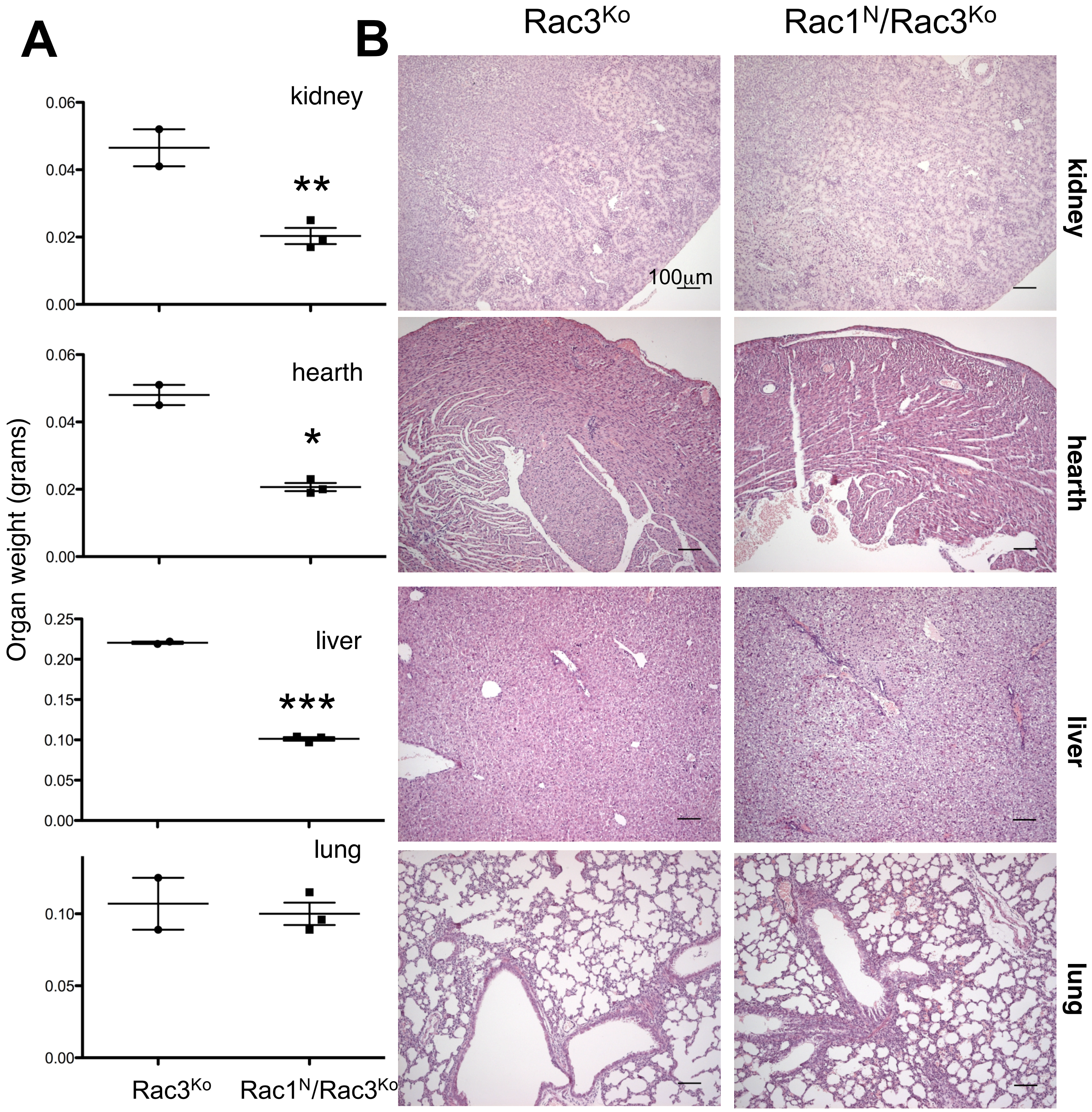

Supporting Information Figure 6. Several vital organs are reduced in size, but have normal histological appearance in double KO mice. P13 Rac3^{KO} and Rac1^{N}/Rac3^{KO} animals were sacrificed and organs were explanted and weighted (**A**). **B**) Cryosections of the indicated organs from P13 Rac3^{KO} and Rac1^{N}/Rac3^{KO} animals were analyzed by hematoxylin and eosin staining Bar: 100µm.

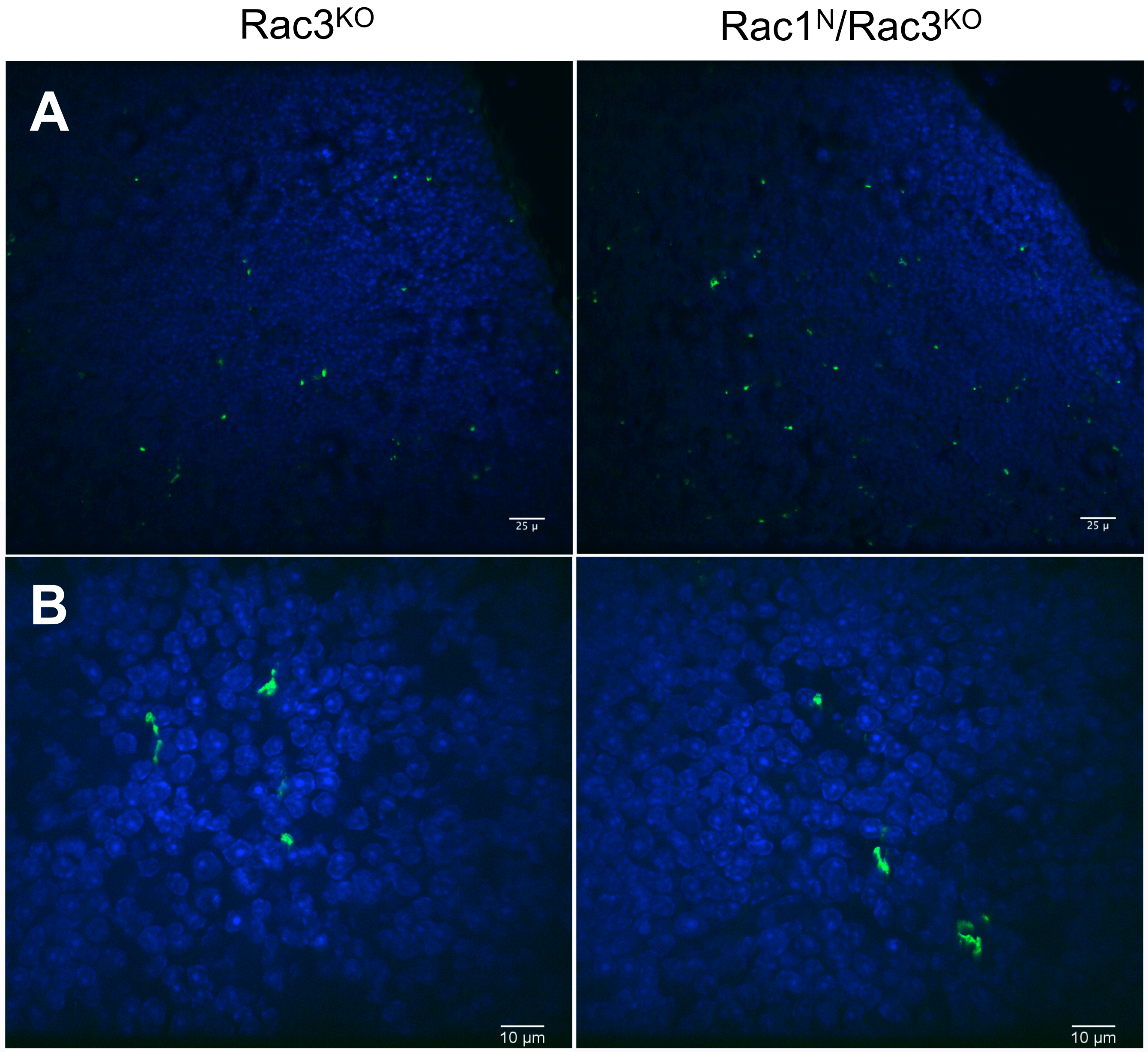

Supporting Information Figure 7. Immunolocalization of TH-positive nerve endings in the thymus. Cryosections of the thymus from P13 Rac3^{KO} and Rac1^{N}/Rac3^{KO} mice were labelled with anti-TH antibody (green) and with DAPI (blue), and analyzed by confocal microscopy. Bars, 10 (**A**) and 25 µm (**B**).

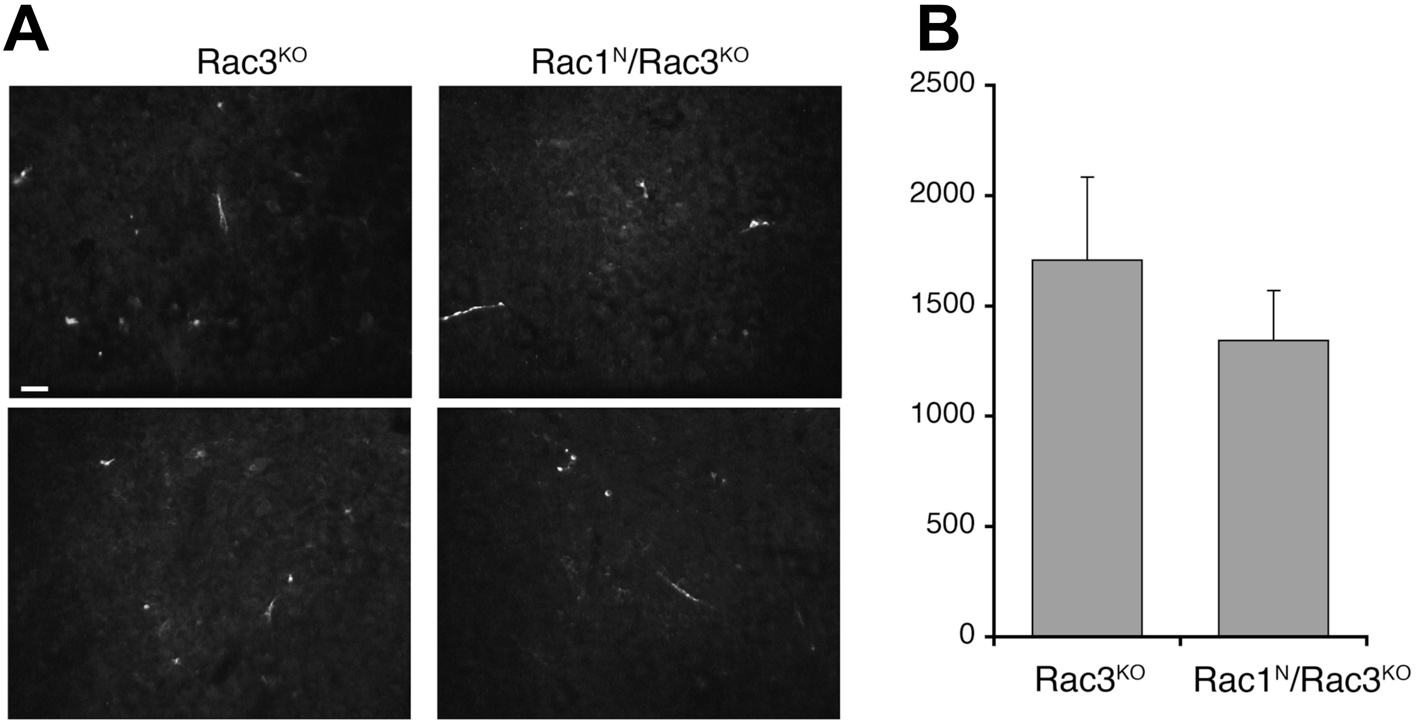

Supporting Information Figure 8. Immunolocalization of neurofilament-positive nerve endings in the thymus. (**A**) Cryosections of the thymus from P13 $Rac3^{KO}$ and $Rac1^{N}/Rac3^{KO}$ mice were labelled with anti-neurofilament antibodies, and analyzed by confocal microscopy. Bar, 25 µm. (**B**) Quantification of the area (in pixels) occupied by neurofilament-positive endings in P13 thymus. Bars are means ± SEM (n=19 fields from three different mice per genotype). P=0.40 (by the Student's *t* test).

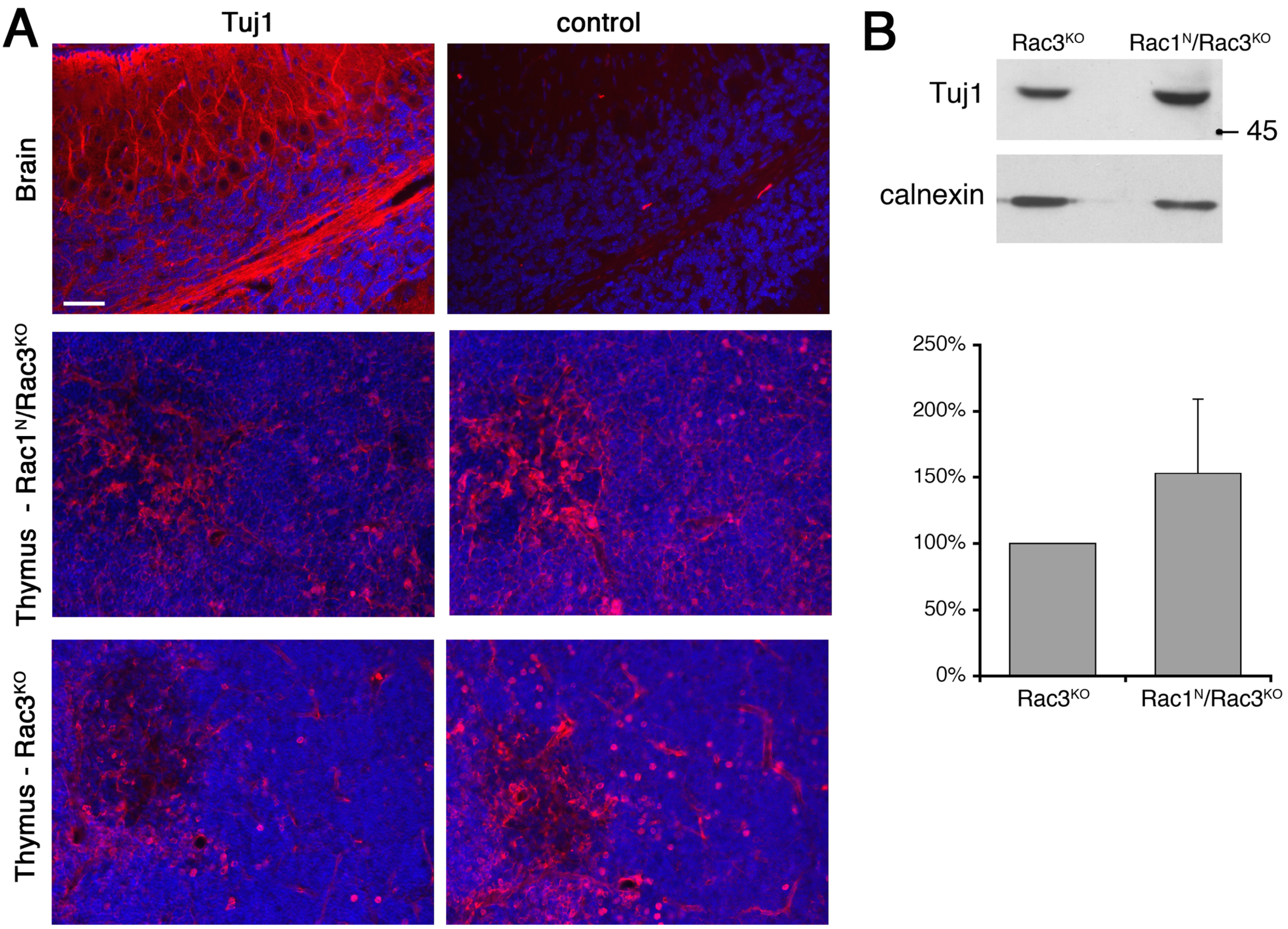

Supporting Information Figure 9. Expression of β3 tubulin in the thymus. (**A**) Cryosections of brain and of thymus from either P13 Rac3^{KO} or Rac1^{N}/Rac3^{KO} mice were immuno-labelled for β 3 tubulin (Tuj1 mAb, red). Nuclei were stained with DAPI (blue). Bar, 50 µm. The exposure times for the brain were 300 msec, those for the thymus were 60 msec. (**B**) Immunoblot (top) and quantification (bottom) of the expression of β3 tubulin using the Tuj1 mAb on lysates from thymi of P13 Rac3^{KO} or Rac1^{N}/Rac3^{KO} mice. Quantification was done after normalization on an internal control (calnexin). Bars are normalized means ± SEM (n=3 mice per genotype). P=0.24 (by the Student's *t* test).

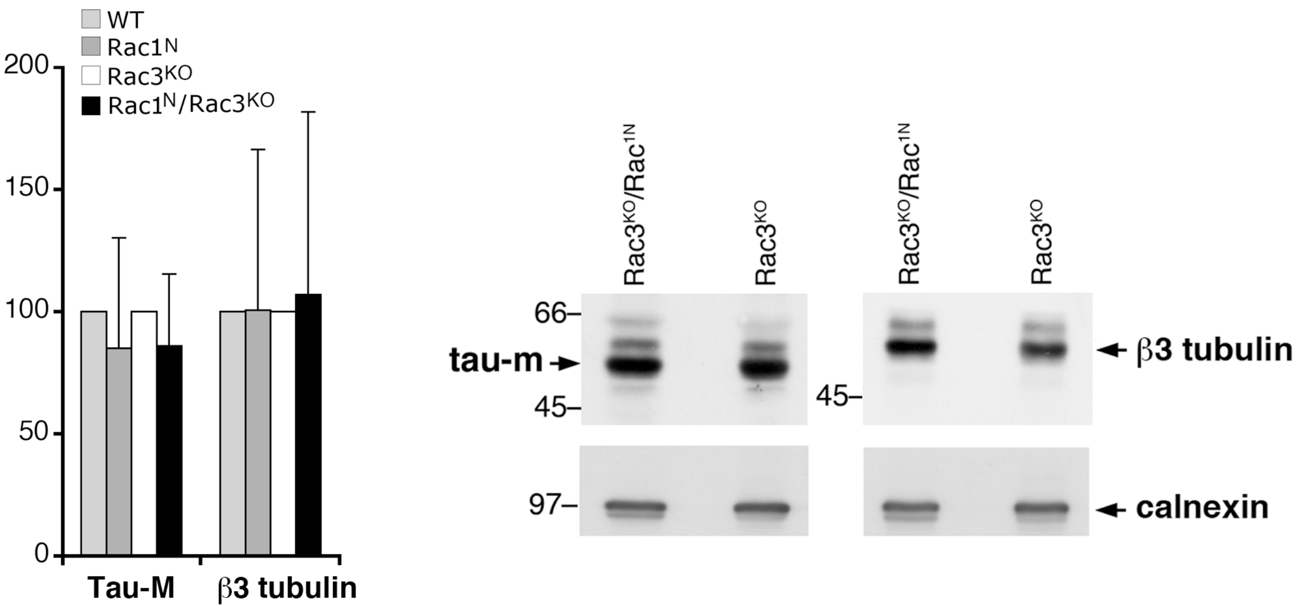

Supporting Information Figure 10. Expression of tau and β3 tubulin in the spleen. Right: immunoblot for tau, β3 tubulin, and calnexin on spleen lysates from double knockout and control mice. Left: for the quantification, the expression of the major intermediate band of tau (tau-M) and of β3 tubulin were normalized with respect to corresponding calnexin levels. Values are presented as normalized means ± SEM (n = 2-5 spleens per mouse genotype).
